# A phase II clinical and pharmacodynamic study of temsirolimus in advanced neuroendocrine carcinomas

**DOI:** 10.1038/sj.bjc.6603419

**Published:** 2006-10-10

**Authors:** I Duran, J Kortmansky, D Singh, H Hirte, W Kocha, G Goss, L Le, A Oza, T Nicklee, J Ho, D Birle, G R Pond, D Arboine, J Dancey, S Aviel-Ronen, M-S Tsao, D Hedley, L L Siu

**Affiliations:** 1 Department of Medical Oncology and Hematology, Princess Margaret Hospital Phase II Consortium, 610 University Avenue, Suite 5-718, Toronto, ON, Canada M5G 2M9; 2 Memorial Sloan Kettering Cancer Center, New York, USA; 3 University of Chicago, Chicago, USA; 4 National Cancer Institute, Bethesda, USA

**Keywords:** mTOR, neuroendocrine carcinoma, phase II, pS6, temsirolimus

## Abstract

Standard cytotoxic treatments for neuroendocrine tumours have been associated with limited activity and remarkable toxicity. A phase II study was designed to evaluate the efficacy, safety and pharmacodynamics of temsirolimus in patients with advanced neuroendocrine carcinoma (NEC). Thirty-seven patients with advanced progressive NEC received intravenous weekly doses of 25 mg of temsirolimus. Patients were evaluated for tumour response, time to progression (TTP), overall survival (OS) and adverse events (AE). Twenty-two archival specimens, as well as 13 paired tumour biopsies obtained pretreatment and after 2 weeks of temsirolimus were assessed for potential predictive and correlative markers. The intent-to-treat response rate was 5.6% (95% CI 0.6–18.7%), median TTP 6 months and 1-year OS rate 71.5%. The most frequent drug-related AE of all grades as percentage of patients were: fatigue (78%), hyperglycaemia (69%) and rash/desquamation (64%). Temsirolimus effectively inhibited the phosphorylation of S6 (*P*=0.02). Higher baseline levels of pmTOR (phosphorylated mammalian target of rapamycin) (*P*=0.01) predicted for a better response. Increases in pAKT (*P*=0.041) and decreases in pmTOR (*P*=0.048) after treatment were associated with an increased TTP. Temsirolimus appears to have little activity and does not warrant further single-agent evaluation in advanced NEC. Pharmacodynamic analysis revealed effective mTOR pathway downregulation.

Neuroendocrine carcinomas (NEC) comprise a family of neoplasms derived from the diffuse neuroendocrine system with a wide range of morphologic, functional and behavioural characteristics (Jensen and Doherty, 2005). Conventional chemotherapy has not shown a significant activity in advanced NEC, except for islet cell carcinomas (ICC) where streptozocin-based combinations with either 5-fluorouracil or doxorubicin have produced partial remissions in 40–60% of selected patients (Moertel *et al*, 1982; Moertel *et al*, 1992). However, carcinoid tumours (CT) seem to be quite chemo-resistant, with response rates of <10% and a median 5-year survival of 18% (Oberg, 2001). Alternative treatments including *α*-interferon 2b and local hepatic therapies for isolated metastases, such as hepatic arterial bland or chemo-embolisation, resection and radiofrequency ablation, have not demonstrated significant impacts on overall survival (OS).

The mammalian target of rapamycin (mTOR) is a serine–threonine kinase that participates in the regulation of cell growth, proliferation and apoptosis through modulation of cell cycle progression (Vignot *et al*, 2005). Mammalian target of rapamycin regulates initiation of cap-dependent translation by phosphorylating 4E-binding protein 1, releasing eukaryotic initiation factor 4E (eIF4E) to bind with eIF4G of the 4E initiation protein complex. Mammalian target of rapamycin also modulates ribosomal function by phosphorylating p70^S6^ kinase which activates the ribosomal protein S6 (Podsypanina *et al*, 2001). Signalling through the PI3K/AKT/mTOR pathway leads to an increase in translation, particularly of proteins regulating cell cycle progression and metabolism. In cancer cells, aberrant activation of the pathway may occur through increased signalling via growth factor receptors, activating mutations/amplification of the pathway kinases or by loss of the tumour suppressor protein PTEN. The latter has been described in NEC (Wang *et al*, 2002). Temsirolimus (sirolimus 42-ester 2,2-bis hydroxymethyl propionic-acid; CCI-779) is a more water-soluble ester derivative of its parent compound sirolimus, selected for development as an anticancer agent based on its more favourable pharmaceutical characteristics and superior therapeutic index. Temsirolimus has already been tested in phase I and II trials with promising activity and good safety profile (Atkins *et al*, 2004; Baselga *et al*, 2004; Raymond *et al*, 2004; Chan *et al*, 2005). In phase I studies, rash and mucositis were dose-limiting, and other adverse events (AE) observed include eczematous reactions, dry skin, herpes-type lesions, mild myelosupression, hypercholesterolaemia and hypertriglyceridemia (Atkins *et al*, 2004; Baselga *et al*, 2004; Raymond *et al*, 2004).

The primary objective of this study was to examine the objective response rate (ORR) of temsirolimus in patients with recurrent or metastatic NEC. Secondary objectives were: (i) to assess drug toxicity; (ii) to determine through pharmacodynamic evaluations whether temsirolimus downregulates mTOR and modulates elements of the PI3K/AKT/mTOR pathway and (iii) to associate pretreatment molecular characteristics, and molecular changes between paired tumour biopsies, with clinical outcome.

## PATIENTS AND METHODS

### Eligibility

Patients were eligible if they were 18 years of age or older and had histologically or cytologically confirmed NEC of either carcinoid or pancreatic ICC pathologies. Patients had to have documented progressive metastatic disease within 6 months of study entry. Previous chemotherapy, investigational agents, radioactive therapies and/or radiation were allowed if completed >4 weeks before study entry. Previous local therapy (e.g. bland or chemo-embolisation) was allowed if completed >6 weeks before study entry. Patients were required to have measurable disease, an ECOG performance status ⩽2, normal serum cholesterol and triglyceride, adequate haematologic, hepatic, renal and cardiac functions and a life expectancy of >3 months. Patients had to have tumour lesions accessible for core biopsy, and must agree to undergo tumour biopsy before and 2 weeks after initiation of temsirolimus.

### Treatment

Temsirolimus at 25 mg was administered as a 30-min intravenous infusion on a weekly schedule. Four weeks of treatment were considered as one cycle.

### Assessment of toxicity

Adverse events were graded using the National Cancer Institute Common Terminology Criteria for Adverse Events v3.0.

### Dose modifications

Dose modifications of temsirolimus were based on haematologic and non-haematologic toxicities at the time of every weekly dose. Upon recovery of toxicity within a maximum delay of 3 weeks, temsirolimus may be re-started with a dose reduction. Stepwise dose modifications from 25 to 20, 15 and 10 mg were allowed, but doses once reduced cannot be re-escalated.

### Response assessment

Radiological imaging was repeated every 8 weeks to assess for tumour response until disease progression, completion of study treatment or discharge of patient from study. Tumour responses were evaluated according to standard RECIST criteria (Therasse *et al*, 2000). Objective responses were confirmed by central independent radiological review.

### Correlative studies

#### Archival tissues

Archival paraffin slides were stained for PTEN, p53, pAKT, pS6 and pmTOR (phosphorylated mTOR) by immunohistochemistry. Slides were pretreated and incubated with primary antibody (Appendix 1), followed by biotin-conjugated secondaries and HRP-Streptavidin labelling reagent (ID Labs Inc., London, Ontario, Canada). Two pathologists, who were blinded to clinical outcome (M-ST. and SA-R), evaluated independently for each marker: (i) the percentage of stained cells, which was converted to a four-tiered system (0=0; 1=⩽10%; 2=11–50% and 3=⩾51%), and (ii) the intensity of staining (0–3). The sum of both values was the specimen's score (range 0–6). The scores from the two pathologists were then averaged. PTEN was scored as either ‘lost’ or ‘retained’.

#### Computerised image analysis for paired tumour biopsies

Tumour biopsies taken before and after 2 weeks post-treatment with temsirolimus were collected into 10% neutral buffered formalin, fixed overnight and transferred in 70% ethanol for processing into paraffin blocks. Four micrometre thick sections were cut onto Surgipath x-tra™ slides. Slides were pretreated by either pepsin digestion or microwave retrieval and then incubated in primary antibody overnight inside a moist chamber (Appendix 1). This was followed with Cy3- or Cy5-conjugated secondaries (Jackson ImmunoResearch, West Grove, PA, USA) for immunofluorescence. Paired biopsies were stained for pAKT, pS6, pmTOR and peIF4G. Biopsy slides were imaged with a laser scanning TISSUEscope (Biomedical Photometrics, Waterloo, Ontario, Canada) using 2 *μ*m per pixel resolution. All images were analysed blinded using MCID Elite software (Imaging Research Inc., St Catharines, Ontario, Canada). A threshold was set to select for positive staining of a specific marker of interest. Results were expressed as the percentage of positively stained areas in square microns within the tumour regions, and the staining intensity reported as mean optical density (IOD) in grey levels.

### Statistical analysis

The primary endpoint was objective tumour response rate (complete response (CR) or partial response (PR)). Secondary endpoints included toxicity, stable disease (SD) rate, response and SD duration, time to progression (TTP) and OS. A two-stage phase II design was used. The treatment combination would be assumed to be inactive if the objective response was at most 5% and active if it was at least 25%, with *α*=0.05 and *β*=0.10. After completion of accrual of 15 evaluable patients to stage I, while no CR or PR was observed, there were 10 patients who fulfilled the criteria for SD. After plotting out patient's pretreatment and post-treatment tumour progression rate, it was observed that at least three out of these 10 patients had a significant decrease in this parameter after starting temsirolimus, along with improvement in their disease-related symptoms. As a result of these findings, it was hypothesised that temsirolimus may have antitumour activity in this tumour type but possibly not reliably evaluated by conventional RECIST criteria. The protocol was amended and additional patients accrued to stage II.

The Kaplan–Meier method was used to estimate survival outcomes. Time to progression was measured from the first date a patient received study medication until the date of tumour progression. Progression-free survival (PFS) was estimated from the first date a patient received study medication until the date of progression, or death; OS was measured from the first date a patient received study medication until the date of death or last date the patient was known to be alive.

Molecular marker levels before temsirolimus treatment and their changes measured on study were investigated as predictors of objective tumour response, TTP and OS, using Spearman rank correlation coefficients and Cox proportional hazards regression. All tests were two-sided and *P*-values of 0.05 or less were considered statistically significant.

This study was a collaborative effort between three consortia, led by the Princess Margaret Hospital Phase II Consortium. Local institutional review board approvals were obtained at all participating centres.

## RESULTS

### Patients

A total of 37 patients were accrued to the study from January 2004 to July 2005. One patient did not receive any treatment owing to progressive disease before treatment initiation and was considerable ineligible. Thirty-six patients received at least one dose of temsirolimus and were evaluated for safety. Patient characteristics are listed in Table 1.

### Efficacy

#### Tumour response

**Figure 1:**
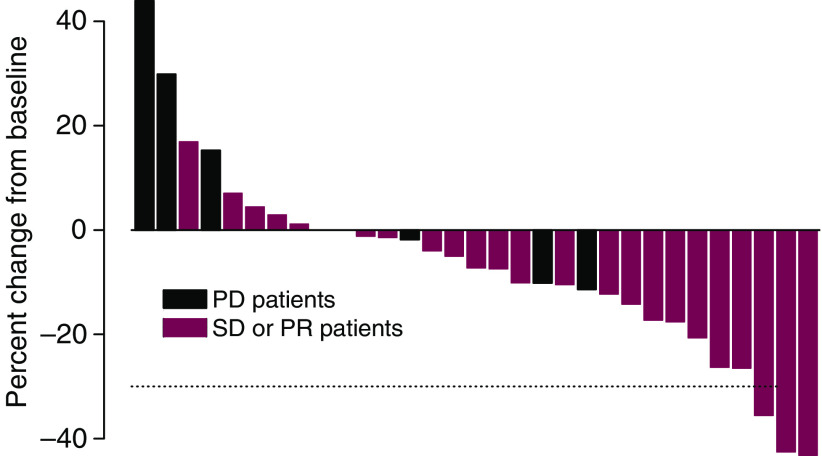
Maximal percentages of tumour reduction for target lesion(s) by RECIST criteria (*Note*: some patients with PD progressed owing to new or increasing non-target lesions, or by symptomatic progression).

Two patients, one with CT and one with ICC, achieved a confirmed PR. One of them progressed after 18 cycles (11.4 months after first observation of PR) and the other came off study owing to unrelated cardiac disease (4.5 months after first observation of PR). A third patient had an unconfirmed PR at the end of cycle 8 and discontinued therapy not owing to toxicity. Twenty additional patients had SD of at least 2 months' duration and among these, 10 patients continued treatment beyond six cycles. Ten patients progressed on temsirolimus without ever achieving an objective response. Eight of these patients had radiological evidence of disease progression, one had symptomatic progression during cycle 1, and one patient died of disease before end of cycle 2. Figure 1 shows the maximum percentage of target tumour lesion(s) reduction compared to baseline as assessed by the RECIST criteria, listed by individual study patients.

As serum markers such as chromogranin A or 5HIAA were not mandated in this protocol and not collected in all patients, the biochemical response could not be assessed.

The intent-to-treat response rate for the entire study cohort is 2/36=5.6% (95% CI 0.6–18.7%) and tumour control (SD+PR) rate is 23/36=63.9% (95% CI 46.2–79.2%). Response outcomes were similar between the CT and ICC histologies, with PR rates of 4.8 and 6.7%, respectively.

#### TTP

**Figure 2:**
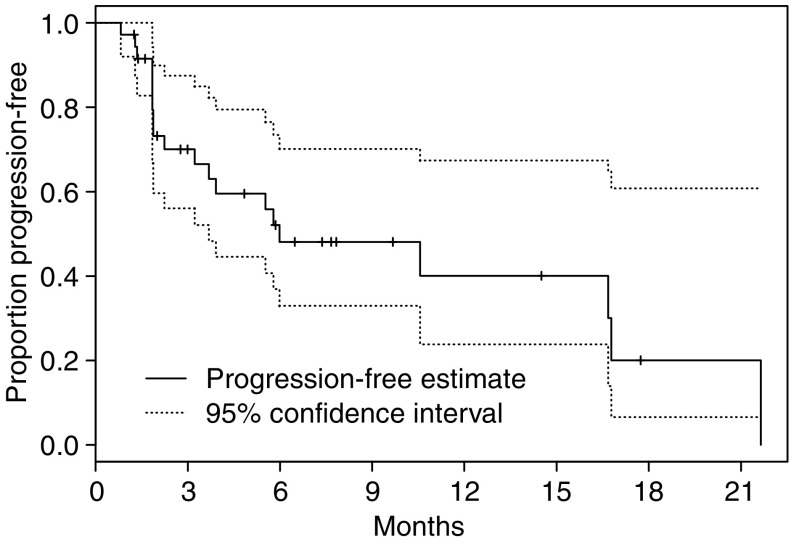
Time to progression for entire study cohort.

Five patients remain on study and continue to receive treatment as of January 2006. Of the 31 patients who have come off treatment, the reasons for discontinuation were: PD (15), symptomatic PD (four), death (one), physician discretion (two), AE (seven) and patient withdrawal (two). Median TTP is estimated to be 6.0 months (95% CI 3.7-not reached); 6-month progression-free rate is estimated to be 48.1% (95% CI 33.0–70.1%) and 1-year progression-free rate is estimated to be 40.1% (95% CI 23.8–67.4%) (Figure 2).

#### Survival

**Figure 3:**
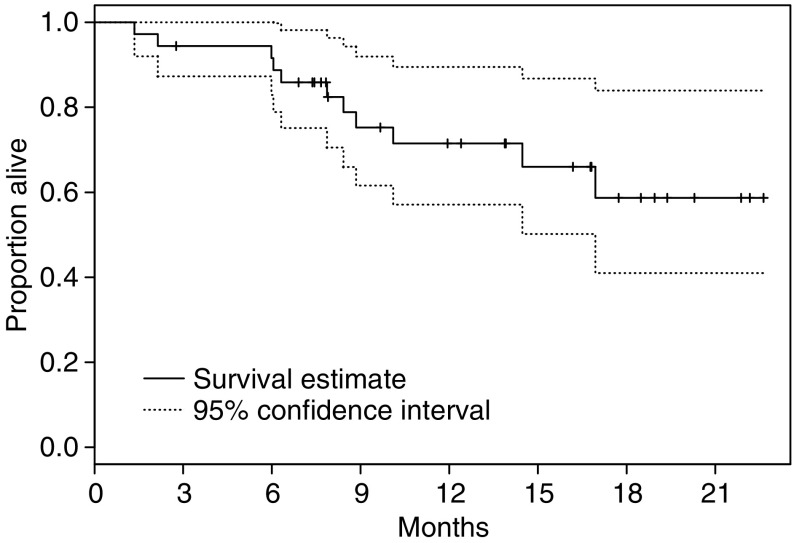
Overall survival for entire study cohort.

At the time of reporting, 11 patients have died. Median follow-up on the 25 patients alive at last follow-up is 13.9 months (range 2.8–22.6 months), minimum follow-up is 6.9 months. Median OS has not been reached; 6-month survival rate is estimated to be 91.6% (95% CI 82.9–100.0%) and 1-year survival rate is estimated to be 71.5% (95% CI 57.1–89.5%) (Figure 3). Patients with ICC appeared to have slightly better TTP and OS but statistical comparisons were not made for this subgroup analysis (Table 2).

### Toxicity

Safety and tolerability data are available for 213 treatment cycles, with a median number of four cycles delivered per patient (range 1–21), AE deemed by the investigator as at least possibly related to study drug and that have occurred in more than 10% of the cycles are shown in Table 3. Overall, treatment with temsirolimus was well tolerated. The most frequent AE of all grades, at least possibly related to study treatment, were: fatigue (78% of patients; 53% of cycles), hyperglycaemia (69% of patients; 54% of cycles) and rash/desquamation (64% of patients; 50% of cycles). Severe AE that were grade ⩾3 and at least possible attribution were uncommon. Seven patients developed pneumonitis considered as possibly related to temsirolimus, three of whom required drug discontinuation. Other observed AE include two patients who had grade 5 events. One died from a pneumothorax and bronchospasm in cycle 2 unlikely related to temsirolimus and the other died from pulmonary embolism 5 days after removal from study, deemed to be possibly drug related.

### Pharmacodynamic analysis

Twenty-two patients had archival specimens evaluable for analysis of baseline molecular markers. No significant association was seen between any of the pretreatment markers tested (PTEN, p53, pS6, pmTOR and pAKT) and tumour response, TTP or survival. Despite being nonstatistically significant, the loss of PTEN expression was associated with a trend towards a shorter TTP (*P*=0.07).

**Figure 4:**
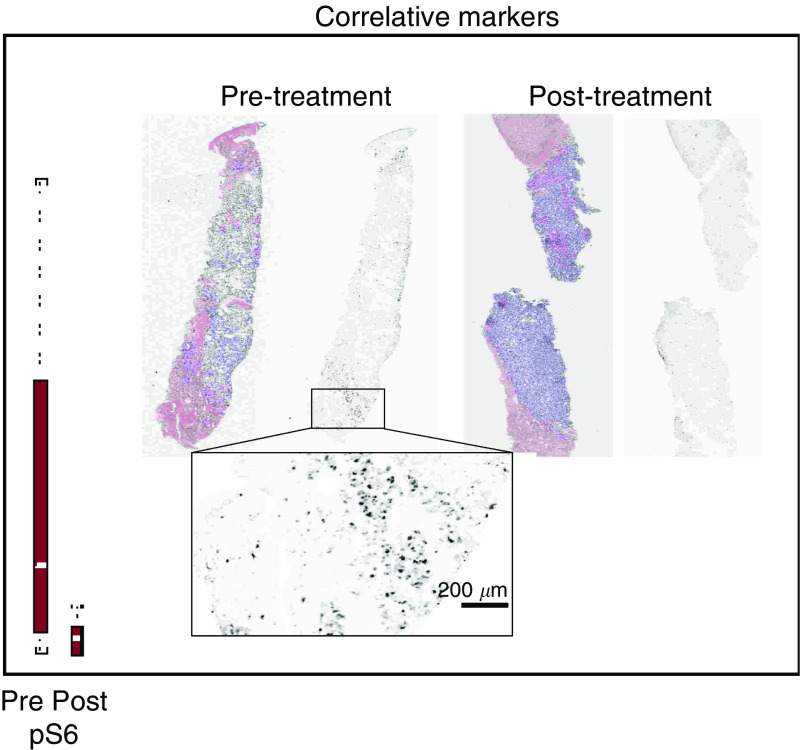
Pre- and post-treatment liver biopsies. Tissue sections were first immunofluorescence-labelled for S235/236-S6 ribosomal protein, imaged, and then restained with H&E. The grey scale images of pS6 are unenhanced, at original resolution.

Paired baseline and post-treatment biopsies were obtained from 23 patients, and 13 paired-samples were evaluable. The best responses of these 13 patients were one PR, eight SD, three PD and one nonevaluable. Baseline expression levels of pAKT, pS6, pmTOR and peIF4G were determined and compared with expression levels following 2 weeks of treatment. Temsirolimus effectively inhibited the phosphorylation of S6 (*P*=0.02) (Figure 4) and higher baseline levels of pS6 showed a trend towards being predictive of a better response (*P*=0.097). Higher baseline levels of pmTOR were predictive of tumour response (*P*=0.01). Increases in the expression of pAKT (*P*=0.041), and decreases in pmTOR (*P*=0.048) after 2 weeks on treatment, were associated with an increased TTP.

A discrepancy was noted in the predictive abilities of pS6 and pmTOR between freshly procured pretreatment specimens *vs* paraffin-embedded archival specimens. However, only seven patients had both archival specimens and pretreatment tumour biopsies that were evaluable. Hence, statistical comparisons were not performed.

## DISCUSSION

Neuroendocrine carcinomas, generally subcategorised into CT and ICC, often pursue an indolent clinical course. However, patients ultimately will become symptomatic either as a result of increasing tumour bulk or hormonal hypersecretion. Somatostatin analogues have proven successful in ameliorating symptoms of the carcinoid syndrome but its benefit in survival is unclear (Saltz *et al*, 1993). Streptozocin and DTIC-based regimens have been tested with only modest activity and may also be associated with significant toxicity (Moertel and Hanley, 1979; Engstrom *et al*, 1984; Moertel *et al*, 1992; Bukowski *et al*, 1994; Rivera and Ajani, 1998; Cheng and Saltz, 1999; Ramanathan *et al*, 2001; McCollum *et al*, 2004; Sun *et al*, 2005). As conventional systemic approaches remain insufficient and highly toxic, there is an obvious need for novel therapies in this tumour population.

The intent-to-treat response rate of 5.6% and median TTP of 6.0 months observed in our study compares favourably with other targeted therapies tested in this tumour population. A recently reported phase II study of gefitinib in 96 patients with progressive NEC (55 with CT and 42 with ICC) revealed a 6-month-PFS of 51% for the former and 28% for the latter. However, objective responses were only seen in one out of 40 patients (2.5%) in the CT group (Hobday *et al*, 2005). A phase II study with the multi-targeted tyrosine kinase inhibitor sunitinib involving 112 patients (41 CT and 61 ICC), reported an ORR of 8.8%, with a median TTP of 40 weeks and a high percentage of SD (Kulke *et al*, 2005). Efficacy varied by tumour histology with an ORR of 2% (1/41) among CT *vs* 13% (8/61) among ICC. In another phase II study where 44 patients with CT were randomised to bevazucimab or pegylated interferon, the former showed superior activity (4/22 *vs* 0/22 confirmed PR), reduction in tumour perfusion and improvement in PFS at 18 weeks (96% *vs* 68%) (Yao *et al*, 2005). However, neither of these two latter trials required evidence of progressive disease before study entry, therefore the patient populations are likely different from ours.

Regarding histology, in our study temsirolimus appears to be slightly more active in ICC than in CT, as reported with other therapies (Moertel and Hanley, 1979; Engstrom *et al*, 1984; Sun *et al*, 2005; Kulke *et al*, 2006). While our small sample size precludes any definitive conclusions, it is possible that CT and ICC will manifest different sensitivities to different targeted agents, as is the situation with cytotoxic agents.

Pharmacodynamic evaluations in paired biopsies, confirmed, for the first time in this patient population, that temsirolimus effectively downregulates the phosphorylation of S6, and that higher baseline levels of pS6 and pmTOR seem to predict for a better response. These results are consistent with those reported by Cho *et al* (2005) which analysed predictive markers of temsirolimus in advanced renal cell carcinomas, confirming the value of pS6 (Cho *et al*, 2005). In this study, we have selected an antibody against pmTOR (ser2448) with high specificity, and this may explain the finding of pmTOR as a predictive marker of response.

**Figure 5:**
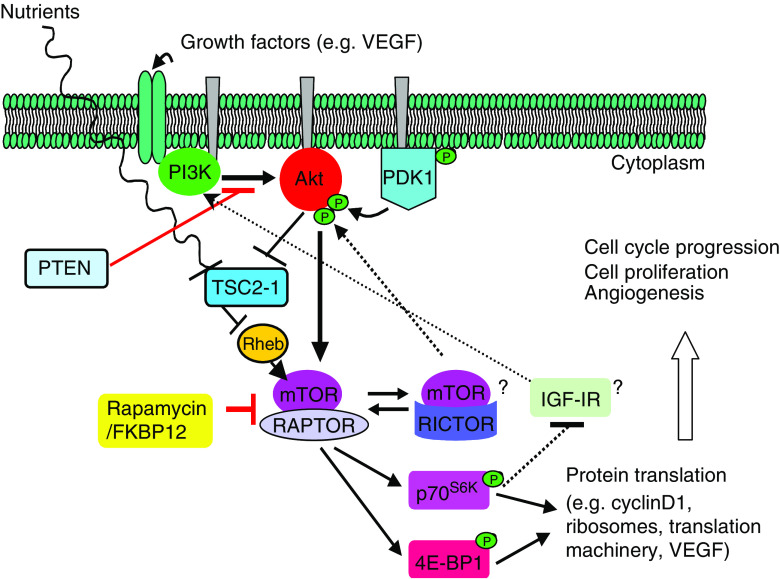
PI3K/AKT/mTOR pathway showing the mTOR protein complexes, mTOR/RAPTOR and mTOR/RICTOR, and the feedback loop involving IGF-IR. Arrows indicate activation; bars indicate inhibition.

Other interesting pharmacodynamic findings include the rising trend in AKT phosphorylation noted after treatment with temsirolimus. Two alternative pathways that induce AKT activation may explain this finding (Figure 5). RAPTOR (regulatory associated protein of mTOR) and RICTOR (rapamycin-insensitive companion of mTOR) are key partnering proteins which complex with mTOR and modulate its functions. Activation of AKT through the mTOR–RICTOR complex could explain our observation (Hresko and Mueckler, 2005; Sarbassov *et al*, 2005). Additionally, AKT phosphorylation has been described through a feedback loop of the PI3K/AKT/mTOR pathway from the insulin-like growth factor I receptor (IGF-IR) (O'Reilly *et al*, 2006). Further pharmacodynamic analysis revealed a positive association between increases in pAKT and decreases in pmTOR with a more prolonged TTP. These exploratory findings require confirmation with larger series. The evaluation of archival specimens searching for prognostic factors only showed a trend towards shorter TTP in those patients with loss of PTEN. The discordance between the results from the archival and the freshly procured pretreatment specimens in our study could be due to differences in the genetic profile of primary and recurrent/metastatic tumours; tumour heterogeneity; and different protocols for specimen handling. Differences in time and speed of tissue fixation after biopsy or resection may significantly affect the phosphorylated states of signalling molecules.

In conclusion, temsirolimus appears to have only modest activity with a manageable toxicity profile in advanced NEC. The results of this study do not warrant further investigation of this drug as a single agent in this patient population. Evaluation of temsirolimus, in combination with other targeted agents, such as a multi-kinase inhibitor or an antiangiogenic compound, should be considered. The loss of PTEN expression could represent a poor prognostic marker for NEC. Pharmacodynamic analysis in paired tumour biopsies reflected effective mTOR pathway downregulation and identified possible predictive factors. Evaluations in larger populations are needed to confirm these findings.

## Figures and Tables

**Table 1:** Patient characteristics

		**Patients *N*=36**	
	**No.**		**%**
*Age (years)*			
Median		56	
Range		36–77	
			
*Sex*			
Female	21		58
Male	15		42
			
*ECOG PS*			
0	16		44
1	19		53
2	1		3
			
*Type of tumour*			
Carcinoid	21		58
Islet cell carcinoma	15		42
			
*Prior treatment*			
Adjuvant chemotherapy	2		6
Palliative chemotherapy	21		58
Radiotherapy	5		14
Bland embolisation	4		11
Surgery	26		72
Octreotide	9		25
			
*No. of prior chemotherapy regimens*			
0	14		39
1	9		25
2	7		19
3	3		8
4	3		8

**Table 2:** Efficacy outcomes by histology

	**Islet cell *n*=15**	**Carcinoids *n*=21**
	***n* (%)**	***n* (%)**
*Best response*		
PR	1 (6.7)	1 (4.8)
SD	9 (60.0)	12 (57.1)
PD	4 (26.7)	6 (28.6)
Non-evaluable	1 (6.7)	2 (9.5)
PR+SD	10 (66.7)	13 (61.9)
		
*Time to progression*		
Median (months)	10.6	6.0
6-month (%)	51.6	45.3
1-year (%)	25.8	45.3
		
*Overall survival*		
Median (months)	Not reached	Not reached
6-month (%)	93.3	90.5
1-year (%)	85.6	60.7
		
*Status at last follow-up*		
Alive	11 (73.3)	14 (66.7)
		
*Receiving treatment*		
	2 (13.3)	3 (14.3)

**Table 3:** Drug-related adverse events occurring in >10% of treatment cycles

	**Grade 1–2**		**Grade 3–4**	
**Adverse event**	**Patients *n* (%)**	**Cycles *n* (%)**	**Patients *n* (%)**	**Cycles *n* (%)**
*Constitutional symptoms*				
Fatigue	28 (78)	112 (53)	0 (0)	0 (0)
				
*Hematologic*				
Anaemia	21 (58)	111 (52)	1 (3)	1 (0.5)
Lymphopenia	19 (53)	112 (53)	1 (3)	2 (1)
Thrombocytopenia	18 (50)	61 (29)	1 (3)	1 (0.5)
Leukocytes	13 (36)	37 (17)	0 (0)	0 (0)
				
*Metabolic*				
Hyperglycaemia	17 (47)	92 (43)	8 (22)	24 (11)
Hypercholesterolaemia	13 (36)	68 (32)	2 (6)	7 (3)
Hypertriglyceridaemia	15 (42)	58 (27)	1 (3)	1 (0.5)
ALT	15 (42)	71 (33)	2 (6)	3 (1)
AST	20 (55)	71 (33)	1 (3)	1 (0.5)
ALP	6 (17)	28 (13)	1 (3)	1 (0.5)
Hypoalbuminaemia	9 (25)	35 (16)	0 (0)	0 (0)
Hypophosphataemia	8 (22)	35 (16)	4 (12)	15 (7)
Creatinine	13 (36)	33 (15)	0 (0)	0 (0)
				
*Gastrointestinal*				
Mucositis	26 (72)	71 (33)	0 (0)	0 (0)
Dysgeusia	10 (28)	47 (22)	0 (0)	0 (0)
Nausea	13 (36)	35 (16)	0 (0)	0 (0)
Diarrhoea	11 (30)	40 (19)	3 (9)	3 (1)
Constipation	10 (28)	28 (13)	0 (0)	0 (0)
Anorexia	13 (36)	31 (14)	0 (0)	0 (0)
				
*Dermatologic*				
Rash (desquamation)	22 (61)	104 (48)	1 (3)	3 (1)
Rash (acneiform)	16 (44)	76 (36)	0 (0)	0 (0)
Dry skin	12 (33)	40 (19)	0 (0)	0 (0)
Pruritus	9 (25)	38 (18)	0 (0)	0 (0)
				
*Pulmonary*				
Pneumonitis	7 (19)	35 (16)	0 (0)	0 (0)

**Table A1:** 

**Antibody**	**Source**	**Pretreatment**	**Dilution**	**IF/IHC**
pAKT ser473	Cell Signaling	Microwave	1:300	IF–Cy5 and IHC
peIF4G ser1108	Cell Signaling	Microwave	1:200	IF–Cy5
pmTOR ser2448	Cell Signaling	Microwave	1:400	IF–Cy5 and IHC
pS6RP ser235/236	Cell Signaling	Pepsin	1:200	IF–Cy5 and IHC
p53 D07	Novo Castra	Microwave	1:100	IHC
PTEN	Cell Signaling	Microwave	1:200	IHC

IF=immunofluorescence; IHC=immunohistochemistry.

## Appendix 1

Antibodies used for immunofluorescence and immunohistochemistry analysis.

Table A1
